# Effects of *Shuhe granules* on chronic pain in patients with rheumatoid arthritis and fibromyalgia: a randomized, double-blind, placebo-controlled trial

**DOI:** 10.3389/fphar.2026.1761852

**Published:** 2026-05-04

**Authors:** Yi-hang Yu, Yu-xi Li, Meng-ling Peng, Guang-yao Zhang, Kai-xin Gao, Hai-fang Du, Mao-jie Wang, Xiao-dong Wu, Wen Xu, Yong-liang Chu, Bi-yun Xu, Run-yue Huang, Zhi-min Yang

**Affiliations:** 1 State Key Laboratory of Traditional Chinese Medicine Syndrome, The Second Affiliated Hospital of Guangzhou University of Chinese Medicine (Guangdong Provincial Hospital of Chinese Medicine), The Second Clinical Medical College of Guangzhou University of Chinese Medicine, Guangzhou, China; 2 Guangdong University of Technology, Guangzhou, Guangdong, China; 3 Guangdong Provincial Key Laboratory of Clinical Research on Traditional Chinese Medicine Syndrome, Guangzhou, China; 4 State Key Laboratory of Dampness Syndrome of Chinese Medicine, The Second Affiliated Hospital of Guangzhou University of Chinese Medicine (Guangdong Provincial Hospital of Chinese Medicine), Guangzhou, China; 5 Zhuhai Branch of the Second Affiliated Hospital of Guangzhou University of Chinese Medicine, Zhuhai, China; 6 Sleep disorder Department The Second Affiliated Hospital of Guangzhou University of Chinese Medicine, Guangzhou, China

**Keywords:** fibromyalgia, pain, randomized controlled trial, rheumatoid arthritis, Shuhe granules

## Abstract

**Background:**

Patients with rheumatoid arthritis (RA) and concomitant fibromyalgia (FM) frequently experience severe, refractory pain, elevated disease activity scores, and substantial psychological distress. Conventional treatments frequently fail to adequately address this complex pain syndrome, and evidence-based traditional Chinese medicine (TCM) interventions for this specific comorbidity remain limited. This pilot study aimed to assess the feasibility of a definitive trial and preliminarily evaluate the efficacy and safety of *Shuhe granules* (SHG), a Chinese herbal formulation, for alleviating chronic pain and associated multidimensional symptoms in this patient population.

**Methods:**

In this randomized, double-blind, placebo-controlled pilot trial, 30 patients diagnosed with both RA and FM were randomly assigned (1:1) to receive either SHG (n = 15) or matching placebo (n = 15) twice daily for 4 weeks, followed by an 8-week follow-up. The primary outcome was the change in pain intensity measured by visual analog scale (VAS). Secondary outcomes included patient and physician global assessments (PGA, PhGA), tender and swollen joint counts (TJC, SJC), FM-specific measures (WPI, SSS, FIQ), and assessments of fatigue, sleep quality, anxiety, and depression. Safety was monitored through adverse event recording and laboratory tests.

**Results:**

Twenty-six patients completed the trial (placebo completers n = 11 vs. SHG n = 15). Linear mixed-effects models showed significant group × time interactions for VAS (*P* < 0.001), PGA and PhGA (*P* < 0.001), and TJC (*P* < 0.001). Within the SHG group, VAS scores showed a clinically meaningful reduction at week 4 (*P* < 0.001), while no significant change occurred in the placebo group during the same period. TJC improvements persisted to week eight in the SHG group (*P* < 0.001). For FM-related outcomes, significant group × time interactions were observed for SSS (*P* < 0.001) and PHQ-9 (*P* < 0.001), with the SHG group achieving clinically meaningful improvements in SSS at week 4 (*P* < 0.001) and significant reductions in PHQ-9 (*P* < 0.001). Although within-group improvements were also observed for FSS and ISI, between-group differences were not statistically significant. No serious adverse events occurred, and laboratory parameters remained within normal ranges for all participants.

**Conclusion:**

This pilot study provides preliminary evidence that SHG may offer short-term benefits in reducing pain and improving physical function, sleep, fatigue, and psychological wellbeing in patients with RA and comorbid FM, with a safety profile comparable to placebo. While these preliminary findings support the potential of SHG as a therapeutic option, they require confirmation in larger, multicenter trials with extended follow-up to establish definitive efficacy and explore underlying mechanisms.

**Clinical Rehabilitation Impact:**

SHG may represent a promising complementary therapy for relieving chronic pain and improving overall health in patients with RA complicated by FM. Larger, multicenter trials with extended follow-up are warranted to confirm these findings, explore underlying mechanisms, and define its role in integrative management strategies.

**Clinical Trial Registration:**

Chinese Clinical Trial Registry (ChiCTR), https://www.chictr.org.cn/showproj.html?proj=221912, Identifier ChiCTR2400081980.18 March 2024.

## 1 Background

Rheumatoid arthritis (RA) is a chronic autoimmune inflammatory disorder characterized by persistent synovitis, progressive cartilage and bone destruction, and eventual disability if inadequately treated (Sahin et al., 2025). Despite considerable advances in pharmacological interventions, including conventional synthetic, biologic, and targeted synthetic disease-modifying anti-rheumatic drugs (DMARDs), pain remains the predominant concern for most patients. Epidemiological data indicate that up to 70% of individuals with RA identify pain relief as their primary therapeutic goal, and approximately one-third develop chronic widespread pain within 5 years of initiating therapy (Andersson et al., 2013).

Fibromyalgia (FM) is a central sensitization syndrome characterized by widespread musculoskeletal pain, fatigue, sleep disturbance, and cognitive dysfunction. It is a frequent comorbidity in RA, with reported prevalence rates ranging from 4.9% to 52.4% (Duffield et al., 2018). The coexistence of FM is termed “fibromyalgic RA”, which represents a distinct clinical phenotype (Scott and Steer, 2007). Compared to patients with RA alone, those with fibromyalgic RA generally exhibit more severe pain, higher disease activity scores, and poorer mental health (Ranzolin et al., 2009; Haliloglu et al., 2014). Notably, 67%–80% of RA patients with high disease activity have comorbid FM, as measured by the Disease Activity Score 28 (DAS28) or Clinical Disease Activity Index (CDAI) (Arnold et al., 2004). These patients also report worse mental component summary scores on the Short Form-36 (SF-36 MCS), poorer sleep quality, higher tender joint counts, and higher scores on the pain DETECT questionnaire (PDQ) (Salaffi et al., 2005).

The coexistence of RA and FM thus poses a significant clinical challenge. Patient-reported outcomes, such as pain and global assessments, are often disproportionately elevated, which can lead to overestimation of disease activity, inappropriate treatment escalation, and unsatisfactory symptom control. This challenge underscores a critical disconnect between objective inflammatory pathology and subjective symptom perception (Minhas et al., 2023). In RA management, swollen joint count serves as a fundamental objective indicator of synovitis and inflammatory activity. However, in patients with fibromyalgic RA, this measure often dissociates from patient-reported pain due to central sensitization (Khot et al., 2024). The persistence of pain despite controlled swelling (indicating inadequate non-inflammatory pain management) or the discordance between high pain scores and low swollen joint counts (suggesting fibromyalgia overlap) both highlight the necessity of evaluating therapeutics through dual lenses: anti-inflammatory efficacy (measured by swollen joint count) and central pain modulation (measured by patient-reported outcomes). Therefore, incorporating swollen joint count alongside pain assessment provides a more comprehensive framework for evaluating treatments targeting this complex comorbidity.

Contemporary clinical management of chronic pain in patients with RA and comorbid FM typically adopts an integrated approach incorporating both pharmacological and non-pharmacological interventions. Conventional pharmacological regimens rely primarily on anti-inflammatory and analgesic agents, including nonsteroidal anti-inflammatory drugs (NSAIDs), glucocorticoids, DMARDs, and biological or targeted synthetic agents (Drosos et al., 2020). Complementary non-pharmacological approaches comprise structured exercise programs, physical therapy modalities, evidence-based psychological interventions, and various Traditional Chinese Medicine (TCM) techniques (Huang et al., 2022). Although conventional pharmacological therapies primarily target inflammatory pathways, they frequently fall short in addressing the multidimensional symptoms of FM, such as fatigue, insomnia, and mood disturbances. Analgesics and central nervous system–acting drugs (e.g., duloxetine, pregabalin) may provide partial benefit but are limited by tolerability, safety concerns, and incomplete efficacy in RA patients with concomitant FM.

Pain relief represents the primary therapeutic goal in FM management, an area where TCM interventions have shown notable potential (Li et al., 2025). In recent years, TCM has gained increasing attention as a complementary therapeutic strategy. For instance, the integration of the TCM-derived metabolite paeoniflorin with conventional biomedicine, supplemented by physical modalities such as hydrotherapy, has been reported to yield a 90% efficacy rate in FM treatment, substantially surpassing outcomes achieved with biomedicine alone (Kong and Wen, 2019). Although several pharmacological agents have been approved for RA or FM as separate conditions, effective strategies for their co-occurrence remain limited. Within the Chinese healthcare system specifically, there is a pronounced lack of methodologically rigorous drug intervention studies and systematized TCM therapeutic strategies explicitly addressing the complex management challenges posed by chronic pain in patients with concurrent RA and FM.

*Shuhe Granules* (SHG), a representative commercial Chinese polyherbal preparation, was formulated based on the extensive clinical expertise of the distinguished TCM physician Yang Zhimin (Gao et al., 2025). This formulation comprises nine distinct botanical drugs (Supplementary Table S1). Originally developed for chronic insomnia and fatigue, SHG has demonstrated potential analgesic and functional benefits in preliminary clinical studies involving patients with immune-mediated conditions.

Current clinical management strategies for RA and FM often remain inadequate for addressing chronic pain, and evidence-supported TCM therapies specifically targeting this comorbidity are notably lacking. In our previous single-arm trial involving RA patients with comorbid insomnia, SHG treatment was associated with statistically significant reductions in pain (measured by Visual Analog Scale, VAS) and disease activity, as well as a significant improvement in Insomnia Severity Index (ISI) scores (Gao et al., 2025). These promising results suggest that SHG may have therapeutic potential in this patient population.

As the first study in China to focus specifically on the comorbidity of RA and FM, this randomized, double-blind, placebo-controlled pilot trial aims to preliminarily evaluate the clinical efficacy and safety of SHG in managing chronic pain in this complex patient group, thereby providing a foundation for a future large-scale definitive trial.

## 2 Materials and methods

### 2.1 Investigational medications

#### 2.1.1 Shuhe granule

##### 2.1.1.1 Composition

“Type A extract” refers to botanical drugs whose extracts are included in national or regional pharmacopoeias and are used as active ingredients in phytopharmaceuticals intended for regulated medical use (licensed, listed, or registered medicines) (Heinrich et al., 2022; Heinrich et al., 2020). As a Type A extract, *Shuhe Granules* (SHG) is composed of nine botanical drugs (Supplementary Material 1, Supplementary Table S1), and was formulated based on the extensive clinical experience of the distinguished TCM physician Yang Zhimin. Through modern pharmaceutical technology, the decoction of SHG is processed by extraction, concentration, drying, and granulation. The daily dosage of SHG comprises the following botanical drugs: *Neolitsea cassia (L.) Kosterm. [*Lauraceae*; Guizhi-branch]*: 15 g; *Paeonia lactiflora Pall. [*Paeoniaceae*; Baishao-radix]*: 15 g; *Zingiber officinale Roscoe [*Zingiberaceae*; Shengjiang-rhizome]*: 18 g; *Glycyrrhiza uralensis Fisch. [*Fabaceae*; Gancao-radix et rhizome]*: 12 g; *Ziziphus jujuba Mill. [*Rhamnaceae*; Dazao-fructus]*: 18 g; *Panax ginseng C. A. Mey. [*Araliaceae*; Renshen-radix et rhizome]*: 10 g; *Angelica sinensis (Oliv.) Diels [*Apiaceae*; Danggui-radix]*: 10 g; *Ophiopogon japonicus (Thunb.) Ker Gawl. [*Asparagaceae*; Maidong-radix]*: 10 g; *Morinda officinalis How [*Rubiaceae*; Bajitian-radix]*: 10 g.

##### 2.1.1.2 Manufacturing process

Qualified botanical drug pieces, verified against prescription specifications, were added to an extraction tank with drinking water and subjected to heat reflux extraction twice. The first extraction used 10 times the amount of water relative to the total material weight and was heated for 1.5 h. The second extraction used 8 times the amount of water and was heated for 1 h. The two filtrates were combined and collected in a settling tank for subsequent processing. This extraction ratio and duration were optimized based on the established process conditions for Shuxin Anshen Paste (SAP) from our team’s previous research, which demonstrated maximal retention of the active constituents from each botanical component (Gao et al., 2025). As SHG is a modified formulation derived from SAP, the same extraction parameters were adopted.

The combined filtrate was transferred to a vacuum concentrator and concentrated to a relative density of 1.030–1.070 (measured at 60 °C), then stored under refrigeration for further use. Separately, maltodextrin was dissolved in two to three volumes of purified water preheated to 80 °C–100 °C with continuous stirring until completely dissolved. The solution was filtered through a 100-mesh sieve, added to the concentrated extract, and mixed evenly. The resulting mixture was heated to 60 °C–70 °C and filtered through a 100-mesh sieve. The filtered extract was subjected to spray drying to achieve a moisture content of 5.0% (rapid determination), and the spray-dried powder was collected.

The SHG spray-dried powder and additional maltodextrin were placed in a three-dimensional motion mixer and blended for 5–10 min to ensure uniformity. The mixed powder was then processed in a dry granulator, and the resulting granules were passed through a 14-mesh sieve. A 10-mesh/60-mesh vortex vibrating sieve was employed to obtain the desired granule size fraction, with qualified powder collected accordingly. Finally, the sieved granules were mixed at 30 Hz for 3–5 min and collected as the finished product.

##### 2.1.1.3 Storage conditions

SHG granules should be stored away from direct sunlight, in a dry environment (relative humidity below 80%), and at a cool temperature (10 °C–30 °C) to prevent degradation, moisture absorption, clumping, or mildew. The product should be kept sealed to avoid contamination by odors or moisture, and should not be stored together with chemical reagents, volatile substances, or toxic materials. The packaging and appearance of the granules should be regularly inspected; if abnormalities such as discoloration, odor, or agglomeration are observed, use should be discontinued and a pharmacist consulted. The sponsor distributes packaging according to random coding, with usage instructions, batch number, manufacturer, and drug number printed on the outer packaging box.

##### 2.1.1.4 Product specification and quality control documentation

SHG (active formulation, batch number 2312309) was manufactured by Jiangyin Tianjiang Pharmaceutical Co., Ltd. under GMP-compliant conditions. Each sachet contains 10.6 g of granules, with an expiration date of November 2026. The product should be stored under sealed conditions. Quality control inspection was performed in accordance with the Chinese Pharmacopoeia (2020 Edition) and the company’s internal quality standards, including tests for solubility, moisture content, and microbial limits. All test results conformed to the established specifications. The quality control documentation was prepared by Wang Shaoling, reviewed by Zhu Caixia, and approved by Gu Lingfeng. The Certificate of Analysis is provided in Supplementary Material 2. The HPLC fingerprint of SHG was established to ensure the consistency and authenticity of the botanical components (Supplementary Material 3). Chemical fingerprint analysis was performed using ultra-performance liquid chromatography coupled with Q-exactive Orbitrap mass spectrometry, revealing over 100 common chemical peaks. Quantification of marker compounds was performed to determine the content of active pharmaceutical ingredients (Supplementary Materials 4, 5). All analytical results complied with the established specifications.

##### 2.1.1.5 Safety considerations

The dosages of all botanical drug components in SHG comply with the specifications of the Chinese Pharmacopoeia, and no clinical reports to date have documented serious adverse reactions associated with the individual botanical drugs in this formulation. Should mild symptoms such as oral ulcers or sore throat occur during treatment, patients may take the granules with light salt water, as these symptoms typically resolve spontaneously within 5–7 days without requiring discontinuation. If symptoms persist beyond 7 days without improvement, patients are advised to seek further evaluation at a specialized TCM outpatient clinic.

#### 2.1.2 Placebo

##### 2.1.2.1 Composition

The placebo granules were prepared using food-grade excipients and were formulated to match SHG in smell, appearance, taste, and weight while containing no therapeutic ingredients. The composition included maltose crystals as the primary bulking agent, which is mainly composed of maltose along with oligosaccharides, disaccharides, and trace amounts of maltotriose and glucose. Lactose was incorporated as an additional filler. For color matching, three food-grade colorants were employed: lemon yellow (soluble yellow powder), sunset yellow (soluble yellow powder), and caramel (soluble brown powder). A bittering agent containing propylene glycol, water, triacetin, bitter substances, and food-grade flavorings was added to replicate the slight bitterness of the active formulation (Supplementary Material 1, Supplementary Table S2).

##### 2.1.2.2 Storage conditions

All raw materials were stored under appropriate conditions, cool, ventilated, and dry environments, with lactose requiring protection from moisture, pressure, and high temperatures, while the bittering agent required sealed storage in a cool, dry place.

##### 2.1.2.3 Manufacturing process

The placebo was prepared using food-grade excipients including maltodextrin, lemon yellow pigment, sunset yellow pigment, caramel pigment, and a bittering agent. These components were blended for 30 min to ensure uniform distribution. The resulting mixture was then subjected to dry granulation to obtain granules within the particle size range of 12–40 mesh.

##### 2.1.2.4 Product specification and quality control documentation

The placebo granules (batch number 2312309) were manufactured by Jiangyin Tianjiang Pharmaceutical Co., Ltd. under GMP-compliant conditions. Each sachet contains 10.6 g of granules, with an expiration date of November 2026. The product should be stored under sealed conditions. Quality control inspection was performed in accordance with the Chinese Pharmacopoeia (2020 Edition) and the company’s internal quality standards, including tests for solubility, moisture content, and microbial limits. All test results conformed to the established specifications. The quality control documentation was prepared by Wang Shaoling, reviewed by Zhu Caixia, and approved by Gu Lingfeng. The Certificate of Analysis for the traditional Chinese medicine formula granules is provided in Supplementary Material 6.

##### 2.1.2.5 Safety considerations

No clinical reports to date have documented serious adverse reactions associated with the placebo. Should mild symptoms such as oral ulcers or sore throat occur during treatment, patients may take the granules with light salt water, as these symptoms typically resolve spontaneously within 5–7 days without requiring discontinuation. If symptoms persist beyond 7 days without improvement, patients are advised to seek further evaluation at a specialized TCM outpatient clinic.

### 2.2 Study design

This study was designed as a single-center, randomized, double-blind, placebo-controlled pilot trial conducted at the Guangdong Provincial Hospital of Traditional Chinese Medicine (TCM) (GPHCM) from March 2024 to June 2025. The trial protocol was approved by the Institutional Ethics Committee of GPHCM (Approval No. BF2024-017-01) and registered in the Chinese Clinical Trial Registry (ChiCTR2400081980). The study adhered to the principles outlined in the Declaration of Helsinki and the Tokyo Declaration for human research, and was reported in accordance with the Consolidated Standards of Reporting Trials (CONSORT) guidelines for randomized controlled trials (Schulz et al., 2010).

### 2.3 Participants

#### 2.3.1 Inclusion criteria

Eligible participants were aged 18–75 years and fulfilled either the 1987 American College of Rheumatology (ACR) classification criteria or the 2010 ACR/European League Against Rheumatism (EULAR) criteria for RA, as well as the 2016 ACR diagnostic criteria for FM. Participants were also required to have a pain visual analogue scale (VAS) score of ≥2 cm.

#### 2.3.2 Exclusion criteria

Exclusion criteria consisted of: 1) severe chronic pain due to other causes (e.g., diabetic neuropathy, postherpetic neuralgia); (2) diagnosis of other severe rheumatic immune diseases; (3) acute or chronic infectious diseases (e.g., hepatitis B or C virus infection, tuberculosis); (4) serious cardiovascular, respiratory, hepatic, renal, or hematopoietic disorders; (5) laboratory abnormalities such as hemoglobin <90 g/L, white blood cell count <3.0 × 10^9^/L, platelet count <100 × 10^9^/L, glomerular filtration rate <40 mL/min, or aspartate aminotransferase (AST) or alanine aminotransferase (ALT) levels >1.5 times the upper limit of normal; (6) significant neurological or psychiatric disorders; (7) pregnancy or lactation; (8) participation in another clinical trial within 4 weeks prior to screening. All participants provided written informed consent before enrollment.

### 2.4 Sample size

This pilot trial aimed to recruit 30 participants, in accordance with the CONSORT extension for pilot and feasibility studies, which recommends 12–30 participants to assess feasibility parameters rather than definitive efficacy (Eldridge et al., 2016). Assuming an expected mean difference in VAS change of 2 points with a standard deviation of 1.5 (Mudano et al., 2019; Bilberg et al., 2024), the estimated effect size (Cohen’s d) was 1.33 (95% CI: 0.78–1.88). To address potential limitations related to the small sample size, we employed Bayesian factor analysis. With a prior distribution set as Cauchy (0, 0.707), the posterior probability indicated that the intervention effect was greater than one point with a probability of 89% [20]. Sensitivity analysis showed that even with a dropout rate of 20% (n = 24), the study could detect an effect size of d > 1.0 with a power of approximately 65%.

### 2.5 Randomization and blinding

#### 2.5.1 Randomization

This trial employed a simple randomization procedure to assign 30 participants in a 1:1 ratio to either the treatment or control group (n = 15 per group). The randomization sequence was generated by the Methodology Team in Clinical Research of Traditional Chinese Medicine at GPHCM using the PROC PLAN procedure in SAS. To ensure allocation concealment, Jiangyin Tianjiang Pharmaceutical Co., Ltd., which was not involved in the trial’s execution, handled the drug packaging and coding based on the randomization schedule. Following the signing of informed consent forms, eligible participants were enrolled and assigned to their respective groups by the attending physicians.

#### 2.5.2 Blinding procedures

##### 2.5.2.1 Blinding and allocation concealment

A two-level blinding system was implemented to ensure robust allocation concealment. Level 1 concealed the actual treatment assignment (definitive blind code), while Level 2 masked the meaning of the group codes (e.g., Group A or B). The sealed code sheets for both levels were prepared in duplicate and securely stored by an independent party.

##### 2.5.2.2 Unblinding procedures

A two-stage unblinding procedure was adopted. First-stage unblinding was performed after database lock and validation, at which point the statistical center provided the biostatistician with group assignments coded as “A” or “B” for interim analysis. Following completion of the statistical analysis and the final study report, second-stage unblinding was conducted during the clinical summary meeting to reveal the actual treatment corresponding to each code.

##### 2.5.2.3 Emergency unblinding

For emergency situations, opaque kraft paper envelopes were prepared, each labeled “Emergency Envelope-Shuhe Granules-Study Number”along with sponsor information and a warning not to open unless necessary. Investigators were permitted to open an envelope only in the event of a serious adverse event (SAE) or urgent medical need, and were required to record their name and the date and time of opening on the envelope. Each envelope contained a sealed slip indicating assignment to either the SHG or placebo group. Emergency envelopes could only be opened after joint evaluation by the principal investigator and the monitor; such cases were treated as dropouts, with adverse event data included in the safety analysis but potentially excluded from the efficacy evaluation. The trial blind would be considered invalid if the entire blinding code was compromised or if the rate of emergency envelope opening exceeded 20%.

To further ensure subject safety, the central randomization system incorporated an emergency unblinding procedure. In cases of SAEs requiring knowledge of the treatment assignment, the investigator could promptly submit an unblinding request through the online system. This request required authorization by an approved physician, typically the principal investigator. Upon approval, the treatment assignment for that specific subject was immediately revealed. Once a subject’s blinding was broken, the participant was considered unblinded and withdrawn from the study without delay. All case report forms (CRFs) and relevant data for unblinded subjects were to be completed and promptly returned to the lead clinical trial unit for inclusion in the final dataset.

### 2.6 Interventions

Both SHG and placebo granules will be provided by the Jiangyin Tianjiang Pharmaceutical Co., Ltd. Patients in the experimental group received SHG, while those in the control group received the placebo. Both were administered orally as two sachets per dose (10.6 g each), twice daily, dissolved in warm water, and taken 1 hour after meals. The treatment period lasted 4 weeks, with a total observation follow-up period of 8 weeks. The placebo granules were designed to match SHG in appearance, taste, odor, and texture, and were confirmed to maintain blinding integrity (Supplementary Materials 7, 8).

### 2.7 Concomitant medications

**FIGURE 1:**
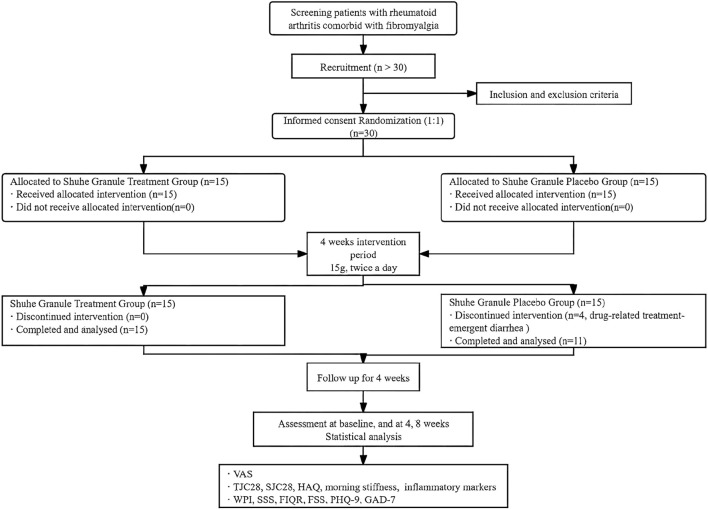
Flow diagram of the study. VAS: Visual Analog Scale; TJC28: tender joint count (28 joints); SJC28: swollen joint count (28 joints); HAQ: Health Assessment Questionnaire; WPI: Widespread Pain Index; FSS: Fatigue Severity Scale; SSS: Symptom Severity Scale; FIQR: Fibromyalgia Impact Questionnaire-Revised; ISI: Insomnia Severity Index; PHQ-9: Patient Health Questionnaire-9; GAD-7: Generalized Anxiety Disorder-7.

Both groups received standardized lifestyle counseling on insomnia management. Participants were allowed to continue stable doses of concomitant medications for chronic comorbidities, including NSAIDs, low-dose glucocorticoids (≤10 mg/day prednisone equivalent), DMARDs, and psychotropic agents, provided these remained unchanged for at least 4 weeks prior to enrollment. Figure 1 displays the flowchart of the study.

## 3 Outcomes and measurements

### 3.1 Primary outcomes

The primary outcome was the change in pain intensity measured by the visual analog scale (VAS) at baseline, week 4, and week 8. The VAS consists of a 10-cm line, with 0 indicating no pain and 10 representing the most intense pain tolerable.

### 3.2 Secondary outcomes

Secondary outcomes included RA disease activity indicators: morning stiffness duration, tender joint count (28 joints) (TJC), swollen joint count (28 joints) (SJC), Health Assessment Questionnaire (HAQ) score, rheumatoid factor (RF), anti-cyclic citrullinated peptide antibody (anti-CCP), C-reactive protein (CRP), erythrocyte sedimentation rate (ESR), and Disease Activity Score 28 (DAS28).

FM-related indicators included: widespread pain index (WPI), symptom severity score (SSS) (Wolfe et al., 2010), revised Fibromyalgia Impact Questionnaire (FIQR) (Salaffi et al., 2023; Bennett et al., 2009), Fatigue Severity Scale (FSS) (Gencay-Can and Can, 2012), Insomnia Severity Index (ISI) (Calandre et al., 2022), Patient Health Questionnaire (PHQ-9), and Generalized Anxiety Disorder seven-item scale (GAD-7) (Ten Brink et al., 2020). Assessments were conducted at baseline, week 4 (end of treatment), and week 8 (follow-up).

### 3.3 Safety assessments

Safety was evaluated through participant self-reported adverse events (AEs) and laboratory testing, including complete blood count, liver function (ALT, AST), and renal function (BUN, serum creatinine), performed at baseline and week 4. All AEs and SAEs were documented in case report forms, with SAEs reported to the Ethics Committee within 24 h. The study sponsor provided coverage for any trial-related injuries.

## 4 Statistics analysis

This study was a prospective randomized controlled pilot trial. For efficacy evaluation, the per-protocol set (PPS) was used as the primary analysis population, defined as participants who completed at least three-quarters of the protocol-specified intervention course. Safety evaluation was conducted on the safety set (SS), comprising all participants who received at least one dose of the study medication. Baseline demographic characteristics, safety indicators, concomitant medications, and adverse reactions were summarized using descriptive statistics and analyzed using chi-square tests, independent samples t-tests, or Mann-Whitney U tests as appropriate. A linear mixed-effects model (LMM) was employed to analyze the repeatedly measured outcomes, including VAS, PGA/PhGA, TJC/SJC, HAQ, RF/CCP/hs-CRP/ESR/DAS28-CRP,WPI/SSS, FIQR, FSS, ISI, PHQ-9, and GAD-7 scores. This model was chosen as it effectively handles missing data and the non-independent data structure inherent in repeated measures, making it particularly suitable for the small sample size of this pilot study. In the model, participant ID was specified as a random effect, while time and group were included as fixed effects, along with the group-by-time interaction term. A two-tailed significance level of α = 0.05 was set for all analyses. Estimated marginal means were used to calculate the scores for each outcome measure for each group at each time point. Post-hoc pairwise comparisons between groups and between time points were adjusted using Bonferroni correction to mitigate the risk of Type I error due to multiple comparisons. Minimal clinically important differences (MCIDs) were used to determine the clinical relevance of observed changes. MCIDs were: ≥15 min reduction for morning stiffness; ≥two to three joints reduction for TJC/SJC; ≥0.22 points reduction for HAQ; ≥20% reduction for RF/CCP/hs-CRP/ESR; and ≥0.6 points reduction for DAS28-CRP; ≥1 point reduction for WPI; ≥0.5 point reduction for SSS; ≥10% reduction for FIQR; ≥0.5 point reduction for FSS; ≥6 points reduction for ISI; ≥5 points reduction for PHQ-9; and ≥4 points reduction for GAD-7. Data were analyzed using SPSS (version 27.0).

## 5 Results

### 5.1 Participant flow and baseline characteristics

Of the 30 randomized participants, 26 completed the trial (placebo completers n = 11 vs. SHG n = 15) per protocol and were included in the primary efficacy analysis (per-protocol set, PPS). Four participants from the placebo group discontinued prematurely: two withdrew after 2 weeks due to diarrhea (resolved upon discontinuation), and two were lost to follow-up for personal reasons unrelated to the study. Following unblinding, all four dropouts were confirmed to be from the placebo group, and their withdrawal was determined to be unrelated to the outcome measures. Baseline characteristics were well balanced between groups, with no significant differences in age, sex, body mass index, or disease duration (all *P* > 0.05; Table 1). All patients received treatment according to their allocation (Figure 1).

**TABLE 1:** Baseline demographic and clinical characteristics.

Characteristics	Placebo (n = 11)	SHG (n = 15)	Statistics	*P*-value
Age (years)	53.36 ± 11.93	54.53 ± 7.45	*t* = −0.308	0.761
Male	1 (9.1%)	0 (0%)	-	0.423
Female	10 (90.9%)	15 (100%)	​	​
BMI (kg/m^2^)	20.50 [18.50, 23.00]	21.80 [18.90, 22.60]	Z = −0.026	0.990
Disease duration (months)	122.91 ± 110.40	85.00 ± 70.23	*t* = 1.071	0.295

Continuous data are presented as mean ± SD, or median [IQR] as appropriate; Categorical data are presented as n (%). BMI: body mass index, SHG: *shuhe granules* treatment.

### 5.2 Primary outcome measures

Linear mixed-effects modeling revealed significant time effects and group × time interactions for VAS (*F* = 13.261, *P* < 0.001), PGA (*F* = 13.335, *P* < 0.001), and PhGA (*F* = 13.335, *P* < 0.001), whereas the main effect of group was not significant (*P >* 0.05) (Table 2), indicating that the temporal trajectories of these outcomes differed between groups.

**TABLE 2:** Fixed effects tests for primary outcome measures.

Characteristics	Fixed effects	*F*	*P*-value
VAS	Intercept	96.203	<0.001^**^
	Group	0.065	0.801
	Time	13.261	<0.001^**^
	Group × time	10.646	<0.001^**^
PGA	Intercept	90.500	<0.001^**^
	Group	0.022	0.884
	Time	13.355	<0.001^**^
	Group × time	16.383	<0.001^**^
PhGA	Intercept	90.500	<0.001^**^
	Group	0.022	0.884
	Time	13.355	<0.001^**^
	Group × time	16.383	<0.001^**^

VAS: visual analog scale; PGA: patient global assessment; PhGA: physician global assessment.

Within-group comparisons, VAS scores in the SHG group decreased rapidly and clinically meaningfully from baseline to Week 4 (mean difference: 1.80 ± 0.35, *P* < 0.001), exceeding the minimal clinically important difference (MCID) of 1.0 point. This improvement was sustained through Week 8 (1.40 ± 0.30, *P* < 0.001). In contrast, the placebo group showed no significant change during the first 4 weeks (*P* = 1.000), with a significant decrease emerging only between Week 4 and Week 8 (1.18 ± 0.28, *P* = 0.001), which just reached the MCID threshold. Consistent patterns were observed for PGA and PhGA (Table 3).

**TABLE 3:** Estimated marginal means and multiple comparisons of primary outcome measures.

Characteristics	Group	Time point	Estimated mean ± SEM	Within-group change (vs. Baseline)	Between-group difference (vs. Placebo)
VAS	Placebo	Week 0	4.64 ± 0.66	—	—
		Week 4	4.91 ± 0.72	0.27 ± 0.41 (*P* = 1.000)	—
		Week 8	3.73 ± 0.72	0.91 ± 0.35 (*P* = 0.048)	—
	SHG	Week 0	5.27 ± 0.56	—	−0.63 ± 0.86 (*P* = 0.472)
		Week 4	3.47 ± 0.62	1.80 ± 0.35 (*P* < 0.001)	1.44 ± 0.95 (*P* = 0.141)
		Week 8	3.87 ± 0.61	1.40 ± 0.30 (*P* < 0.001)	−0.14 ± 0.95 (*P* = 0.884)
PGA	Placebo	Week 0	4.64 ± 0.70	—	—
		Week 4	4.91 ± 0.72	0.27 ± 0.40 (*P* = 1.000)	—
		Week 8	3.73 ± 0.75	0.91 ± 0.35 (*P* = 0.048)	—
	SHG	Week 0	5.40 ± 0.60	—	−0.76 ± 0.92 (*P* = 0.413)
		Week 4	3.47 ± 0.62	1.93 ± 0.34 (*P* < 0.001)	1.44 ± 0.95 (*P* = 0.141)
		Week 8	4.00 ± 0.64	1.40 ± 0.30 (*P* < 0.001)	−0.27 ± 0.99 (*P* = 0.785)
PhGA	Placebo	Week 0	4.64 ± 0.70	—	—
		Week 4	4.91 ± 0.72	0.27 ± 0.40 (*P* = 1.000)	—
		Week 8	3.73 ± 0.75	0.91 ± 0.35 (*P* = 0.048)	—
	SHG	Week 0	5.40 ± 0.60	—	−0.76 ± 0.92 (*P* = 0.413)
		Week 4	3.47 ± 0.62	1.93 ± 0.34 (*P* < 0.001)	1.44 ± 0.95 (*P* = 0.141)
		Week 8	4.00 ± 0.64	1.40 ± 0.30 (*P* < 0.001)	−0.27 ± 0.99 (*P* = 0.785)

VAS: visual analog scale; PGA: patient global assessment; PhGA: physician global assessment; SEM: Standard Error of the Mean. Within-group change represents reduction from baseline (intragroup differences were calculated as baseline minus 4 weeks of treatment/8 weeks of follow-up, positive value indicates improvement after treatment/follow-up). Between-group difference represents placebo minus SHG (positive value favors SHG). *P* values were adjusted using Bonferroni correction.

Between-group comparisons, no significant differences were observed between groups at baseline (all *P* > 0.05). At Week 4, the SHG group showed a greater reduction in VAS compared with placebo (mean difference: 1.44 ± 0.95), but this difference did not reach statistical significance (*P* = 0.141), although the magnitude exceeded the MCID threshold. By Week 8, between-group differences were no longer clinically meaningful (all *P* > 0.05) (Table 3).

### 5.3 RA disease related indicators

Linear mixed-effects models were applied to assess changes in RA-related outcomes, with MCID thresholds predefined for each measure. Significant group × time interactions were observed for TJC (*F* = 10.767, *P* < 0.001) and DAS28-CRP (*F* = 10.344, *P* = 0.004), while marginal interactions were noted for SJC (*F* = 3.033, *P* = 0.067) and HAQ (*F* = 3.188, *P* = 0.059). No significant time effects or interactions were detected for morning stiffness, inflammatory markers (RF, anti-CCP, hs-CRP, ESR), or the main group effects for any measure (all *P* > 0.05) (Table 4).

**TABLE 4:** Fixed effects tests for RA disease related indicators.

Characteristics	Fixed effects	*F*	*P*-value
Morning stiffness, min	Intercept	13.252	0.001^*^
	Group	0.695	0.413
	Time	2.360	0.116
	Group × time	2.514	0.102
TJC	Intercept	25.409	<0.001^**^
	Group	1.244	0.276
	Time	6.762	0.005^*^
	Group × time	10.767	<0.001^**^
SJC	Intercept	6.904	0.015^*^
	Group	0.677	0.419
	Time	1.124	0.341
	Group × time	3.033	0.067
HAQ	Intercept	4.112	0.054
	Group	0.017	0.899
	Time	0.466	0.633
	Group × time	3.188	0.059
RF (IU/mL)	Intercept	18.467	<0.001^**^
	Group	1.004	0.326
	Time	0.346	0.562
	Group × time	0.040	0.844
CCP (U/mL)	Intercept	49.992	<0.001^**^
	Group	0.220	0.643
	Time	1.969	0.173
	Group × time	0.055	0.817
Hs-CRP (mg/L)	Intercept	4.414	0.046^*^
	Group	0.351	0.559
	Time	2.382	0.136
	Group × time	1.834	0.188
ESR (mm/h)	Intercept	103.346	<0.001^**^
	Group	2.068	0.163
	Time	0.027	0.870
	Group × time	1.743	0.199
DAS28-CRP	Intercept	170.198	<0.001^**^
	Group	1.066	0.312
	Time	0.756	0.393
	Group × time	10.344	0.004^*^

RA: rheumatoid arthritis; TJC: tender joint count; SJC: swollen joint count; HAQ: health assessment questionnaire; RF: rheumatoid factor; CCP: cyclic citrullinated peptide; hs-CRP: high-sensitivity C-reactive protein; ESR: erythrocyte sedimentation rate; DAS28-CRP: Disease Activity Score using 28 joints with C-reactive protein.

Within-group changes, no statistically significant or clinically meaningful changes were observed in any RA-related measure throughout the study period in the placebo group (all *P* > 0.05; all changes below MCID thresholds). In the SHG group, TJC showed significant reductions from baseline to Week 4 (3.07 ± 0.52, *P* < 0.001) and baseline to Week 8 (1.93 ± 0.37, *P* < 0.001). The Week 0–4 reduction exceeded the MCID of two to three joints, indicating clinically meaningful improvement. Morning stiffness showed a significant reduction at Week 4 (4.87 ± 1.67, *P* = 0.022), but the magnitude was below the MCID threshold. SJC decreased significantly at Week 4 (0.80 ± 0.27, *P* = 0.020), also below the MCID. Among inflammatory markers, only hs-CRP showed a significant reduction at Week 4 (−1.72 ± 0.77, *P* = 0.036), which was below the 20% MCID threshold. DAS28-CRP increased significantly from baseline to Week 4 (0.30 ± 0.10, *P* = 0.004). No significant changes were observed for HAQ or other inflammatory markers (Table 5).

**TABLE 5:** Estimated marginal means and multiple comparisons of RA disease related indicators.

Characteristics	Group	Time point	Mean ± SEM	Within-group change (vs. Baseline)	Between-group difference (vs. Placebo)
Morning stiffness, min	Placebo	Week 0	11.36 ± 6.14	—	—
		Week 4	11.09 ± 5.45	0.27 ± 1.94 (*P* = 1.000)	—
		Week 8	8.36 ± 5.49	3.00 ± 2.57 (*P* = 0.764)	—
	SHG	Week 0	19.20 ± 5.26	—	−7.84 ± 8.08 (*P* = 0.342)
		Week 4	14.33 ± 4.67	4.87 ± 1.67 (*P* = 0.022)	−3.24 ± 7.18 (*P* = 0.656)
		Week 8	15.60 ± 4.70	3.60 ± 2.20 (*P* = 0.345)	−7.24 ± 7.22 (*P* = 0.326)
TJC	Placebo	Week 0	5.73 ± 2.49	—	—
		Week 4	6.36 ± 2.19	−0.64 ± 0.61 (*P* = 0.916)	—
		Week 8	5.64 ± 2.23	0.09 ± 0.44 (*P* = 1.000)	—
	SHG	Week 0	10.93 ± 2.13	—	−5.21 ± 3.28 (*P* = 0.126)
		Week 4	7.87 ± 1.87	3.07 ± 0.52 (*P* < 0.001)	−1.50 ± 2.88 (*P* = 0.606)
		Week 8	9.00 ± 1.91	1.93 ± 0.37 (*P* < 0.001)	−3.36 ± 2.93 (*P* = 0.262)
SJC	Placebo	Week 0	1.64 ± 1.56	—	—
		Week 4	1.82 ± 1.35	−0.18 ± 0.31 (*P* = 1.000)	—
		Week 8	1.64 ± 1.41	0.00 ± 0.30 (*P* = 1.000)	—
	SHG	Week 0	3.60 ± 1.33	—	−1.96 ± 2.05 (*P* = 0.347)
		Week 4	2.80 ± 1.16	0.80 ± 0.27 (*P* = 0.020)	−0.98 ± 1.78 (*P* = 0.585)
		Week 8	3.33 ± 1.20	0.27 ± 0.25 (*P* = 0.904)	−1.70 ± 1.85 (*P* = 0.368)
HAQ	Placebo	Week 0	0.16 ± 0.17	—	—
		Week 4	0.30 ± 0.21	−0.14 ± 0.07 (*P* = 0.234)	—
		Week 8	0.25 ± 0.20	−0.09 ± 0.06 (*P* = 0.354)	—
	SHG	Week 0	0.33 ± 0.14	—	−0.17 ± 0.22 (*P* = 0.437)
		Week 4	0.23 ± 0.18	0.11 ± 0.06 (*P* = 0.301)	0.07 ± 0.27 (*P* = 0.799)
		Week 8	0.24 ± 0.17	0.09 ± 0.05 (*P* = 0.205)	0.00 ± 0.26 (*P* = 0.974)
RF (IU/mL)	Placebo	Week 0	69.73 ± 32.55	—	—
		Week 4	72.00 ± 33.38	−2.27 ± 8.87 (*P* = 0.800)	—
	SHG	Week 0	111.67 ± 27.87	—	−41.94 ± 42.85 (*P* = 0.337)
		Week 4	116.27 ± 28.59	−4.60 ± 7.60 (*P* = 0.550)	−44.27 ± 43.95 (*P* = 0.324)
CCP (U/mL)	Placebo	Week 0	104.86 ± 23.85	—	—
		Week 4	101.10 ± 23.67	3.76 ± 3.48 (*P* = 0.292)	—
	SHG	Week 0	118.95 ± 20.42	—	−14.09 ± 31.40 (*P* = 0.658)
		Week 4	116.27 ± 20.27	2.68 ± 2.98 (*P* = 0.378)	−15.17 ± 31.17 (*P* = 0.631)
Hs-CRP (mg/L)	Placebo	Week 0	6.54 ± 3.80	—	—
		Week 4	6.66 ± 3.70	−0.11 ± 0.90 (*P* = 0.902)	—
	SHG	Week 0	2.84 ± 3.25	—	3.70 ± 5.00 (*P* = 0.466)
		Week 4	4.56 ± 3.17	−1.72 ± 0.77 (*P* = 0.036)	2.10 ± 4.87 (*P* = 0.670)
ESR (mm/h)	Placebo	Week 0	40.77 ± 8.82	—	—
		Week 4	49.18 ± 9.04	−8.41 ± 8.60 (*P* = 0.338)	—
	SHG	Week 0	63.07 ± 7.56	—	−22.29 ± 11.62 (*P* = 0.067)
		Week 4	56.53 ± 7.74	6.53 ± 7.36 (*P* = 0.384)	−7.35 ± 11.90 (*P* = 0.542)
DAS28-CRP	Placebo	Week 0	2.68 ± 0.34	—	—
		Week 4	2.85 ± 0.37	−0.18 ± 0.11 (*P* = 0.135)	—
	SHG	Week 0	3.39 ± 0.29	—	−0.71 ± 0.45 (*P* = 0.126)
		Week 4	3.09 ± 0.31	0.30 ± 0.10 (*P* = 0.004)	−0.24 ± 0.48 (*P* = 0.628)

RA: rheumatoid arthritis; TJC: tender joint count; SJC: swollen joint count; HAQ: health assessment questionnaire; RF: rheumatoid factor; CCP: cyclic citrullinated peptide; hs-CRP: high-sensitivity C-reactive protein; ESR: erythrocyte sedimentation rate; DAS28-CRP: Disease Activity Score using 28 joints with C-reactive protein. Within-group change represents change from baseline (intragroup differences were calculated as baseline minus 4 weeks of treatment/8 weeks of follow-up, positive value indicates improvement after treatment/follow-up); Between-group difference represents placebo minus SHG (positive value favors SHG). *P* values were adjusted using Bonferroni correction. Data for RF, CCP, hs-CRP, and ESR, were collected only at baseline and Week 4.

Between-group comparisons, no statistically significant or clinically meaningful differences were observed between groups for any RA-related measure at all time points (all *P* > 0.05; all between-group differences below MCID thresholds) (Table 5).

### 5.4 FM-related measures

Linear mixed-effects models were used to evaluate changes in FM-specific outcomes, with predefined MCID thresholds. Highly significant time and group × time interaction effects were observed for SSS (*F* = 38.340 and 29.187, respectively; both *P* < 0.001). PHQ-9 also demonstrated a significant group × time interaction (*F* = 18.489, *P* < 0.001). Marginally significant interactions were noted for WPI (*F* = 2.886, *P* = 0.075) and FSS (*F* = 2.556, *P* = 0.099). FIQR showed a significant time effect (*F* = 3.852, *P* = 0.035), while ISI and GAD-7 showed significant time effects but no interactions (Table 6).

**TABLE 6:** Fixed effects tests for FM disease related indicators.

Characteristics	Fixed effects	*F*	*P*-value
WPI (0–19)	Intercept	1420.792	<0.001^**^
	Group	0.196	0.662
	Time	2.886	0.075
	Group × time	2.886	0.075
SSS (0–12)	Intercept	598.550	<0.001^**^
	Group	0.123	0.729
	Time	38.340	<0.001^**^
	Group × time	29.187	<0.001^**^
FIQR (0–100)	Intercept	122.153	<0.001^**^
	Group	0.324	0.574
	Time	3.852	0.035^*^
	Group × time	0.661	0.525
FSS	Intercept	212.637	<0.001^**^
	Group	0.067	0.798
	Time	1.531	0.237
	Group × time	2.556	0.099
Insomnia (ISI)	Intercept	181.732	<0.001^**^
	Group	0.019	<0.001^**^
	Time	5.685	0.010^*^
	Group × time	0.550	0.584
Depression (PHQ-9)	Intercept	176.136	<0.001^**^
	Group	0.392	0.537
	Time	5.154	0.014^*^
	Group × time	18.489	<0.001^**^
Anxiety (GAD-7)	Intercept	59.721	<0.001^**^
	Group	1.523	0.229
	Time	3.680	0.040^*^
	Group × time	0.443	0.647

FM: fibromyalgia; WPI: widespread pain index; FSS: fatigue severity scale; SSS: symptom severity scale; FIQR: Fibromyalgia Impact Questionnaire-Revised; ISI: insomnia severity index; PHQ-9: Patient Health Questionnaire-9; GAD-7: Generalized Anxiety Disorder-7.

Within-group changes, no statistically significant changes meeting MCID thresholds were observed in the placebo group. Significant reductions in SSS (Week 0–8: 0.73 ± 0.26, *P* = 0.030) were below the 0.5-point MCID. In the SHG group, clinically meaningful improvements were observed in WPI and SSS. WPI decreased significantly from baseline to Week 4 (1.00 ± 0.28, *P* = 0.004), meeting the one-point MCID. SSS showed substantial reductions from baseline to Week 4 (1.67 ± 0.14, *P* < 0.001) and baseline to Week 8 (1.40 ± 0.22, *P* < 0.001), both exceeding the 0.5-point MCID. Although significant improvements were also observed in FIQR (Week 0–8: 9.01 ± 3.27, *P* = 0.033), FSS (Week 0–4: 11.20 ± 3.98, *P* = 0.029), ISI (Week 0–8: 4.40 ± 1.52, *P* = 0.023), and PHQ-9 (Week 0–4: 3.27 ± 0.49, *P* < 0.001; Week 0–8: 2.20 ± 0.57, *P* = 0.002), the magnitudes of change were below their respective MCID thresholds. No significant changes were observed in GAD-7 (Table 7).

**TABLE 7:** Estimated marginal means and multiple comparisons of FM disease related indicators.

Characteristics	Group	Time point	Mean ± SEM	Within-group change (vs. Baseline)	Between-group difference (vs. Placebo)
WPI (0–19)	Placebo	Week 0	9.18 ± 0.44	—	—
		Week 4	9.18 ± 0.36	(-5.551E-16) ± 0.33 (*P* = 1.000)	—
		Week 8	9.18 ± 0.44	(-4.885E-15) ± 0.38 (*P* = 1.000)	—
	SHG	Week 0	10.00 ± 0.38	—	−0.82 ± 0.58 (*P* = 0.173)
		Week 4	9.00 ± 0.31	1.00 ± 0.28 (*P* = 0.004)	0.18 ± 0.47 (*P* = 0.702)
		Week 8	9.20 ± 0.38	0.80 ± 0.33 (*P* = 0.066)	−0.02 ± 0.59 (*P* = 0.975)
SSS (0–12)	Placebo	Week 0	5.82 ± 0.36	—	—
		Week 4	5.55 ± 0.33	0.27 ± 0.17 (*P* = 0.357)	—
		Week 8	5.09 ± 0.40	0.73 ± 0.26 (*P* = 0.030)	—
	SHG	Week 0	6.67 ± 0.31	—	−0.85 ± 0.48 (*P* = 0.089)
		Week 4	5.00 ± 0.28	1.67 ± 0.14 (*P* < 0.001)	0.55 ± 0.43 (*P* = 0.221)
		Week 8	5.27 ± 0.34	1.40 ± 0.22 (*P* < 0.001)	−0.18 ± 0.52 (*P* = 0.740)
FIQR (0–100)	Placebo	Week 0	25.36 ± 4.41	—	—
		Week 4	21.93 ± 3.91	3.43 ± 2.47 (*P* = 0.535)	—
		Week 8	21.85 ± 2.85	3.52 ± 3.82 (*P* = 1.000)	—
	SHG	Week 0	30.27 ± 3.77	—	−4.90 ± 5.80 (*P* = 0.406)
		Week 4	25.14 ± 3.35	5.13 ± 2.12 (*P* = 0.070)	−3.21 ± 5.15 (*P* = 0.539)
		Week 8	21.25 ± 2.44	9.01 ± 3.27 (*P* = 0.033)	0.60 ± 3.75 (*P* = 0.875)
FSS	Placebo	Week 0	40.91 ± 4.73	—	—
		Week 4	43.36 ± 5.13	−2.46 ± 4.65 (*P* = 1.000)	—
		Week 8	39.27 ± 5.21	1.64 ± 4.35 (*P* = 1.000)	—
	SHG	Week 0	49.07 ± 4.05	—	−8.16 ± 6.22 (*P* = 0.202)
		Week 4	37.87 ± 4.40	11.20 ± 3.98 (*P* = 0.029)	5.50 ± 6.76 (*P* = 0.424)
		Week 8	41.07 ± 4.46	8.00 ± 3.72 (*P* = 0.126)	−1.79 ± 6.86 (*P* = 0.796)
Insomnia (ISI)	Placebo	Week 0	13.82 ± 1.57	—	—
		Week 4	12.82 ± 1.64	1.00 ± 1.36 (*P* = 1.000)	—
		Week 8	10.46 ± 1.75	3.36 ± 1.77 (*P* = 0.208)	—
	SHG	Week 0	14.53 ± 1.34	—	−0.72 ± 2.06 (*P* = 0.732)
		Week 4	11.67 ± 1.40	2.87 ± 1.16 (*P* = 0.064)	1.15 ± 2.15 (*P* = 0.598)
		Week 8	10.13 ± 1.50	4.40 ± 1.52 (*P* = 0.023)	0.32 ± 2.30 (*P* = 0.890)
Depression (PHQ-9)	Placebo	Week 0	8.82 ± 1.11	—	—
		Week 4	9.73 ± 1.02	−0.91 ± 0.57 (*P* = 0.372)	—
		Week 8	8.64 ± 1.00	0.18 ± 0.66 (*P* = 1.000)	—
	SHG	Week 0	10.07 ± 0.95	—	−1.25 ± 1.46 (*P* = 0.401)
		Week 4	6.80 ± 0.87	3.27 ± 0.49 (*P* < 0.001)	2.93 ± 1.34 (*P* = 0.039)
		Week 8	7.87 ± 0.86	2.20 ± 0.57 (*P* = 0.002)	0.77 ± 1.32 (*P* = 0.564)
Anxiety (GAD-7)	Placebo	Week 0	5.82 ± 1.58	—	—
		Week 4	3.55 ± 1.04	2.27 ± 1.23 (*P* = 0.227)	—
		Week 8	3.73 ± 0.96	2.09 ± 1.39 (*P* = 0.435)	—
	SHG	Week 0	7.53 ± 1.35	—	−1.72 ± 2.08 (*P* = 0.417)
		Week 4	5.67 ± 0.89	1.87 ± 1.05 (*P* = 0.263)	−2.12 ± 1.37 (*P* = 0.133)
		Week 8	4.87 ± 0.82	2.67 ± 1.19 (*P* = 0.103)	−1.14 ± 1.26 (*P* = 0.375)

FM: fibromyalgia; WPI: widespread pain index; FSS: fatigue severity scale; SSS: symptom severity scale; FIQR: Fibromyalgia Impact Questionnaire-Revised; ISI: insomnia severity index; PHQ-9: Patient Health Questionnaire-9; GAD-7: Generalized Anxiety Disorder-7. Within-group change represents reduction from baseline (intragroup differences were calculated as baseline minus 4 weeks of treatment/8 weeks of follow-up, positive value indicates improvement after treatment/follow-up). Between-group difference represents placebo minus SHG (positive value favors SHG). *P* values were adjusted using Bonferroni correction.

Between-group comparisons, no clinically meaningful between-group differences were detected at any time point, as all mean differences were below MCID thresholds. A statistically significant difference was observed for PHQ-9 at Week 4 (2.93 ± 1.34, *P* = 0.039), but this did not meet the five-point MCID threshold (Table 7).

### 5.5 Safety and AEs

No serious adverse events were observed, and no participants withdrew due to adverse reactions. Laboratory evaluations, including hematology, liver function, and renal function tests, remained stable and within normal ranges in both groups, with no clinically significant abnormalities detected (Table 8). The types and dosages of concomitant medications remained unchanged throughout the study period in both groups. DMARDs were the most frequently prescribed, with usage rates of 72.7% in the placebo group and 60.0% in the SHG group. Targeted small-molecule agents were used slightly more often in the placebo group (46.7% vs. 36.4%), whereas psychiatric medications were more common in the SHG group (26.7% vs. 9.1%), and gastric protectants were prescribed more frequently in the placebo group (45.5% vs. 13.3%). However, none of these differences reached statistical significance (all *P* > 0.05). A detailed comparison of medication use is provided in Table 9. Regarding safety, three adverse events (diarrhea) were reported in the placebo group (27.3%), while no adverse events occurred in the SHG group. Although the between-group difference did not achieve statistical significance (*P* = 0.063), this trend suggests a favorable safety profile for SHG.

**TABLE 8:** Comparison of safety laboratory parameters between groups before and after intervention and adverse events.

Parameter	Placebo (n=11) Baseline	Placebo (n=11) Week 4	SHG (n=15) Baseline	SHG (n=15) Week 4
WBC (×10^9^/L)	5.93 ± 1.47	5.84 [4.90, 7.64]	5.33 ± 1.45	5.31 (4.49,6.03)
Hb (g/L)	121.73 ± 11.65	122.55 ± 12.51	122.47 ± 9.82	120.60 ± 10.60
PLT	265 [231, 411]	310.82 ± 106.19	261 [243, 276]	259.20 ± 47.57
Urine RBC (/μL)	4.00 [0.60, 14.08]	1.30 [0.00, 3.50]	2.60 [0.00, 9.20]	3.00 [0.00, 5.30]
Urine WBC (/μL)	4.70 [2.60, 27.70]	4.00 [1.30, 10.20]	4.00 [1.30, 15.20]	4.00 [1.30, 10.60]
ALT (U/L)	15.00 [0.00, 24.00]	18.00 [15.00, 25.00]	15.00 [13.00, 25.00]	17.00 [12.00, 25.00]
AST (U/L)	20.00 [16.00, 29.00]	21.00 [19.00, 25.00]	22.00 [20.00, 24.00]	22.00 [18.00, 25.00]
BUN (mmol/L)	4.95 [4.33, 5.77]	4.76 ± 1.59	5.16 [4.42, 6.16]	4.91 ± 1.78
Cr (μmol/L)	59.64 ± 12.58	60.27 ± 14.15	61.73 ± 7.42	64.20 ± 8.94

Data presented as mean ± SD (normally distributed) or median [IQR] (non-normally distributed); WBC: white blood cells; Hb: Hemoglobin; PLT: platelets; ALT: alanine aminotransferase; AST: aspartate aminotransferase; BUN: blood urea nitrogen; Cr: Creatinine; RBC: red blood cells; WBC: White blood cells (in urine).

**TABLE 9:** Comparison of medication use and adverse events between groups.

Category	Placebo (n = 11)	SHG (n = 15)	*P*-value
Antihypertensives	2 (18.2%)	0 (0%)	0.169
Lipid-lowering drugs	1 (9.1%)	2 (13.3%)	1.000
NSAIDs	3 (27.3%)	3 (20.0%)	1.000
Corticosteroids	2 (18.2%)	1 (6.7%)	0.556
DMARDs	8 (72.7%)	9 (60.0%)	0.683
Biologics	1 (9.1%)	2 (13.3%)	1.000
Targeted small molecules	4 (36.4%)	7 (46.7%)	0.701
Psychotropic drugs	1 (9.1%)	4 (26.7%)	0.356
Gastroprotective agents	5 (45.5%)	2 (13.3%)	0.095
Bone metabolism drugs	3 (27.3%)	2 (13.3%)	0.620
Calcium supplements	4 (36.4%)	5 (33.3%)	1.000
Adverse events	3 (27.3%)	0 (0.0%)	0.063

Data presented as number of patients (percentage); Fisher’s exact test was used for between-group comparisons; No significant differences were found between groups (all *P* > 0.05); NSAIDs: Non-steroidal anti-inflammatory drugs; DMARDs: Disease-modifying antirheumatic drugs.

## 6 Discussions

The management of chronic pain in patients with RA complicated by FM presents significant clinical challenges, as highlighted by various recent studies (Zhao et al., 2019; Wolfe and Michaud, 2004; Jiao et al., 2016; Arnold, 2009). Emerging evidence suggests that TCM interventions may offer benefits for managing systemic pain in this population (Cai et al., 2023; Pan et al., 2019). In China, millions of RA patients are affected, yet FM is often overlooked. Therefore, there is an urgent need for effective strategies to alleviate chronic pain and improve quality of life in patients with this comorbidity.

Existing evidence indicates that TCM, particularly non-pharmacological modalities, can provide meaningful benefits for patients with RA (Xing et al., 2020; Huang et al., 2015; He et al., 2014). For instance, Juan Bi Pill combined with methotrexate has been shown to reduce disease activity, alleviate arthritic symptoms, and improve quality of life in patients with active RA (Jia et al., 2025). A recent meta-analysis of four randomized controlled trials (RCTs, n = 384) demonstrated that combining non-pharmacological TCM interventions (e.g., acupuncture, moxibustion, or Chinese exercises) with conventional therapy significantly alleviated pain both at post-intervention (VAS reduction, *P* < 0.01) and at 12 months (*P* < 0.05), and also improved Fibromyalgia Impact Questionnaire (FIQ) scores (*P* < 0.05) (Cai et al., 2023). Our pilot trial of SHG similarly provided preliminary evidence consistent with these findings. After 4 weeks, SHG produced a clinically and statistically significant reduction in pain (VAS) from baseline, exceeding the MCID. Significant within-group improvements were also observed in patient and physician global assessments (PGA, PhGA). For RA activity-related measures, notable improvements were seen in TJC and HAQ. Moreover, TJC showed a significant group-by-time interaction, indicating a differential treatment effect over time. Some benefits, including TJC and HAQ, persisted to Week 8. In contrast, between-group differences in VAS attenuated by this time point. These findings suggest early analgesic effects with partial durability beyond the treatment period, particularly for objective joint measures. These findings address a recognized care gap in “fibromyalgic RA,” a phenotype characterized by higher pain burden and worse patient-reported outcomes, where conventional intensification of DMARDs may be ineffective when pain is not primarily inflammatory. By targeting multidimensional symptomatology without escalating immunosuppression, SHG may complement existing RA regimens in this comorbid population.

It has been reported that the impact of mental health on RA disease activity, noting that psychological factors such as anxiety, depression, and sleep disturbances can contribute to overall pain severity (Lwin et al., 2020). A previous study has showed that lifetime rates of major depression were significantly higher in RA + FM versus FM (76.7% and 40%, respectively, *P* < 0.003), as well as the rates of panic disorder (50% and 15%, respectively, *P* < 0.003) (Alciati et al., 2021). Moreover, patients with RA and FM experienced more fatigue (88.1% vs. 50.6%, *P* < 0.001) and had higher anxiety (10 vs. 4, *P* < 0.001) and depression scores (12 vs. 6, *P* < 0.001) compared with patients without FM (Gao et al., 2022). In the present study, beyond joint metrics, SHG was associated with significant improvements in several health domains closely linked to central sensitization. These included sleep disturbance (ISI), fatigue (FSS), and psychological distress (PHQ-9, GAD-7). However, it should be noted that for FSS and ISI, these improvements were observed primarily as within-group changes, whereas between-group differences did not reach statistical significance. In contrast, broader symptom reduction was evident in WPI and SSS, along with functional gains on the FIQR, with SSS showing a significant group-by-time interaction and clinically meaningful improvement. These findings align with the trial’s *a priori* rationale that addressing non-inflammatory drivers of pain may translate into a better overall disease experience in RA with comorbid FM. Given the bidirectional relationships among poor sleep, fatigue, mood, and heightened pain perception in FM, these concurrent changes are coherent with a multimodal effect profile. They may help explain the early VAS response and the sustained functional benefits observed after treatment cessation. The pattern of secondary outcomes points to plausible biopsychosocial mechanisms relevant to FM and central pain amplification. Although the current study was not designed for mechanistic inference, the convergence across pain, function, sleep, and mood outcomes supports continued evaluation of SHG as an adjunctive therapy. Such an approach could reduce reliance on analgesics or unnecessary DMARD escalation in appropriately selected patients.

Regarding safety, comparative analysis showed that the incidence of adverse events in the placebo group was 27.3% (3 of 11 participants), while no adverse events were reported in the SHG group. Despite this numerical difference, the between-group comparison did not reach statistical significance (*P* = 0.063). All adverse events were mild cases of diarrhea, which resolved spontaneously within 1 week without medical intervention. Given that the placebo contained lactose as a filler, the diarrhea may be attributed to lactose intolerance rather than any pharmacological effect. In contrast, SHG is composed of nine botanical drugs, all of which are within the dosage limits specified in the Chinese Pharmacopoeia, with no documented serious adverse reactions in clinical reports. Regarding general safety, laboratory parameters remained stable within normal ranges throughout the study period in both groups, including complete blood count, liver function (ALT, AST), and renal function (BUN, creatinine). Concomitant medication use was well balanced between groups, with no significant differences in the use of DMARDs, biologics, targeted small molecules, or psychotropic agents. No drug-drug interactions were observed during the trial. Overall, the safety profile of SHG was favorable and comparable to placebo. The absence of adverse events in the treatment group, coupled with stable laboratory findings, supports the tolerability of SHG in this patient population.

Several limitations of this study warrant consideration. First, as a single-center pilot trial with a small sample size, the statistical power was limited, and the findings may not be generalizable to broader populations. Second, the intervention period was relatively short, and the follow-up duration was insufficient to assess the long-term efficacy, durability of response, or potential delayed adverse events. Third, the study relied primarily on patient-reported outcomes without incorporating objective biomarkers of central sensitization or neuroimaging, which might have strengthened mechanistic interpretation. Additionally, despite careful placebo design, the possibility of partial unblinding due to taste or sensory differences inherent to herbal formulations cannot be fully excluded.

Future studies should employ larger, multicenter designs with extended treatment and follow-up periods to validate these findings and establish optimal dosing regimens. Incorporating mechanistic endpoints such as quantitative sensory testing, actigraphy-based sleep measures, or inflammatory and neurochemical biomarkers would provide deeper insights into therapeutic pathways. Comparative studies against active pharmacological agents used in FM, such as duloxetine or pregabalin, would further clarify the relative benefits of SHG. Finally, rigorous standardization of botanical drug preparations and detailed reporting of placebo fidelity are essential to enhance reproducibility and ensure alignment with international clinical trial standards.

## 7 Conclusion

In this study, SHG preliminarily demonstrated significant short-term efficacy in alleviating pain and improving physical function, sleep, fatigue, and psychological wellbeing in patients with RA complicated by FM, with a favorable safety profile comparable to placebo. These findings highlight the potential of SHG as a complementary therapeutic approach addressing both inflammatory and non-inflammatory dimensions of pain in this complex patient population. Given the exploratory nature of this pilot trial, with its small sample size, single-center design, and limited treatment duration, these results should be interpreted with caution. Larger, multicenter trials with longer follow-up, rigorous quality control, and mechanistic exploration are warranted to confirm the clinical value of SHG and clarify its role in integrative management strategies for RA with comorbid FM.

## Data Availability

The original contributions presented in the study are included in the article/supplementary material, further inquiries can be directed to the corresponding authors.
